# Medical care in clinical genetics: an experience of decentralization in southern Brazil

**DOI:** 10.31744/einstein_journal/2021AO5708

**Published:** 2021-06-02

**Authors:** Kevin Francisco Durigon Meneghini, Simone de Menezes Karam, Victor Francis Pereira Madruga, Andrea Schulz Silva Ungaretti, Eduarda Cecilia Pinguello, Rafaely Severo

**Affiliations:** 1 Universidade Federal do Rio Grande Faculdade de Medicina Hospital Universitário Dr. Miguel Riet Corrêa Jr. Rio Grande do SulRS Brazil Hospital Universitário Dr. Miguel Riet Corrêa Jr., Faculdade de Medicina, Universidade Federal do Rio Grande, Rio Grande do Sul, RS, Brazil.

**Keywords:** Genetics, medical, Health services research, Epidemiology, Public Health, Hospitals, university

## Abstract

**Objective:**

To describe the population assisted in a genetics outpatient clinic, in a medium-sized town, with respect to diagnosis, type of inheritance, and local impact of genetic care.

**Methods:**

Medical records and genetic consultation forms from 2006 to 2018 were reviewed. The variables analyzed were age, sex, origin, current residence, reason for consultation, professional who requested evaluation, final diagnosis, additional exams and their results.

**Results:**

A total of 609 patients were seen, 65.9% aged 0 to 12 years. Genetic syndromes were suspected in 15.1%, and 11% presented developmental delay. Neurogenetic disorders stood out among adults. Mendelian inheritance was more prevalent (17.8%). Requests for genetic consultation have doubled in the last 5 years, with 44.4% due to suspected genetic syndrome.

**Conclusion:**

Genetic consultations have shown to be an important tool for inpatient care, reducing the waiting time to initiate treatment, attenuating potential associated costs, and guiding the families of patients. Outpatient care provided diagnosis and genetic counseling for users from the city and surrounding region, decreased costs and offered a training environment in medical genetics.

## INTRODUCTION

Within the Unified Health System (SUS - *Sistema Único de Saúde*) the Ordinance 199/2014 by the Ministry of Health established the policy to care for rare diseases (RD), providing the structuring of care from prevention to rehabilitation. According to the World Health Organization (WHO), rare diseases are those that affect 65/100,000 individuals.^(^^1^^)^ Genetic diseases can affect up to 73 per thousand individuals.^(^^2^^)^ Congenital anomalies, mostly determined by genetic causes, affect 5% of newborns.^(^^3^^)^ These conditions have a great impact on affected individuals and their families, because they lead to debilitating physical conditions and may involve several members of the same family, resulting in disability, unemployment, and discrimination.^(^^4^^)^

The search for a definitive diagnosis is often very long, leading to even more suffering.^(^^5^^)^ In contrast, Brazil has 305 physicians specialized in medical genetics, equivalent to a ratio of 0.15 specialist per 100 thousand inhabitants, and 60% of them working in the Southeast Region.^(^^6^^)^ In most states, geneticists still work in capital cities.^(^^7^^)^

## OBJECTIVE

To describe the population assisted in a genetics outpatient clinic, in a medium-sized town, with respect to diagnosis, type of inheritance, and local impact of genetic care.

## METHODS

A cross-sectional, retrospective study was conducted, based on review of medical records from the Medical Genetics Outpatient Clinic, located in Rio Grande, as well as on genetics consulting forms, between 2006 and 2018, excluding a 3-year period (2011, 2012, and 2013), and totaling up 10 years delivering care. During the withdrawal period, some patients were discharged or referred to another service.

Rio Grande, site of the present study, is a municipality of 2,709km^2^ located in the southern region of Rio Grande do Sul State, 350km away from the capital city Porto Alegre. It has approximately 208 thousand inhabitants, 96% of them residing in the urban area.^(^^8^^)^ The main economic activity is port services, fishery and oil refining.^(^^8^^)^ The city is home to the *Universidade Federal do Rio Grande* (FURG) and its *Hospital Universitário Dr. Miguel Riet Corrêa Júnior* (HU-FURG), which provides care exclusively to the SUS, is a reference for 22 cities in the region. The HU-FURG has provided genetic care since 2006.

The target population comprised children and adults seen at the outpatient clinic during the period of study. A standardized form was used for the review with the following variables: age, sex, origin, current place of residence, reason for referral or hospital consultation, professional responsible for the referral, final diagnosis, tests required for the investigation, and their results. All data were tabulated in Excel and classified according to the type of inheritance, and conclusive or non-conclusive diagnosis. Patients who had at least one consultation and, until the moment of concluding data collection, were still waiting for complementary tests and/or other specialized evaluations for conclusion, and unaffected individuals who sought pre-conception genetic counseling due to parental age, consanguinity, or other reasons, were considered under investigation. The classification “non-genetic” was given to cases in which a genetic disease was suspected but was not confirmed, after clinical and laboratory investigation. Data were collected by undergraduate students, and reviewed and analyzed together with the principal investigator. The Medical Genetics Outpatient Clinic has a specific protocol for the first consultation, in which the medical history and family history with pedigree were recorded, including at least three generations, prenatal, delivery, and neonatal history, as well as vaccination records, developmental milestones, use of medications, and laboratory and/or imaging tests already performed. A general physical examination, weight and height measurements, and dysmorphological examination were performed. Whenever indicated, a neurological examination was performed.

According to what was confirmed at the end of the consultation, or during the course of the investigation, subsequent appointments were recorded in follow-up protocols, that is, for Turner syndrome, Down syndrome, dominant cerebellar ataxia, phacomatosis, intellectual disability, among others. Such protocols consider the individual’s progression and anticipatory guidance. During the study period, the following tests were available for investigation at the HU-FURG outpatient clinic: radiographs, ultrasonography, echocardiography, electrocardiography, electroencephalography, and computed tomography, in addition to clinical laboratory. Chromosome karyotyping by G-banding (GTG) and polymerase chain reaction (PCR) for some mutations were performed in a laboratory contracted by the hospital. Inborn errors of metabolism (metabolic screening and enzyme activity dosage) were checked through the network Rede EIM Brasil), at the *Hospital de Clínicas de Porto Alegre* (HCPA) and the *Universidade Federal do Rio Grande do Sul* (UFRGS). Molecular cytogenetic tests (fluorescence *in situ* hybridization - FISH - and microarray-based comparative genomic hybridization (array-CGH) were performed through temporary collaboration in a research project with the *Rede Brasileira de Referência e Informação em Síndromes de Microdeleção* (REDEBRIM/HCPA/UFRGS) [Brazilian Network of Reference and Information on Microdeletion Syndromes]. Next-generation sequencing was not conducted because it was not available in the public health system.

The initial management was based on clinical information, and the hypotheses were investigated until the means available, whether locally or through collaboration with other centers, were exhausted. When the suspicion of a genetic condition persisted, the patient continued to undergo clinical evaluation every six months or annually.

During two different periods, and again since early 2018, karyotype tests and PCR molecular techniques were no longer available. Investigation for familial cancer was never performed. Cases that met criteria for a hereditary cancer predisposition syndrome were referred to the Screening Outpatient Clinic of the *Hospital de Clínicas de Porto Alegre*, through the Municipal Health Secretariat.

The same standardized form used to evaluate the outpatient consultations was used to evaluate the clinical genetics consultations during the same period. The term “consulting” refers to the genetic evaluation of patients admitted to HU-FURG, requested by other specialists. The clinical judgement and the available tests already described were the tools used for evaluation.

The project was approved by the Research Ethics Committee for the Field of Health (CEPAS-FURG), opinion 4212100, CAAE:93280418.3.0000.5324.

## RESULTS

In the period studied, 609 patients were seen at the Medical Genetics Outpatient Clinic. Three quarters of them (78.6%) were from the city where the service was located, 98.4% were urban dwellers, and 65.9% were aged zero and 12 years. More than half were female (Table 1). Part of the patients was referred from private practices; neurologists (n=25) were the specialists who referred most, followed by pediatricians (n=24), and obstetrician-gynecologists (n=7).

**Table 1:** Origin, age, and sex of patients

Origin		n (%)
Municipality of Rio Grande		479 (78.6)
	Others	130 (21.4)
Origin of the referral		
	Pediatrics	111 (18.2)
	Neurologist	58 (9.5)
	Private medical office	56 (9.1)
	APAE	34 (6.6)
	UBS and UBSF	31 (5.0)
	Spontaneous/Family member	29 (4.7)
	Another geneticist	6 (0.9)
	Others	284 (46.6)
Age, year		
	<1	134 (22.0)
	1-4	107 (17.5)
	5-11	161 (26.4)
	12-19	86 (14.1)
	≥20	115 (18.8)
	Not informed	5 (0.8)
Sex		
	Female	333 (54.6)
	Male	276 (45.4)
Total		609 (100)

APAE: Association of Parents and Friends of the Handicapped; UBS: Primary Health Care Unit; UBSF: Family Health Unit.

Regarding referrals for genetic consultation (Table 2), the “suspicion of an unspecified syndrome” stood out (15.1%), which concerned referrals with no description of details related to development, behavior, or morphological alterations, without indicating the suspicion of a classic syndrome. In second place was the neuropsychomotor developmental delay, associated or not with dysmorphisms and/or behavioral changes (11%). Approximately 6% of referrals belonged to the genetic counseling group, in which retrospective and prospective counseling were included.

**Table 2:** Most frequent reasons for referral to genetic consultation

Reasons	n (%)
Suspected unspecified syndrome	92 (15.1)
NPDD associated or not with dysmorphisms and/or behavioral changes	67 (11.0)
Genetic counseling*	35 (5.7)
Isolated or associated school difficulty	31 (5.0)
Suspected autistic spectrum disorder	20 (3.2)
Suspected Down syndrome	20 (3.2)
Suspected neurogenetic disorder†	18 (2.9)
Repeat abortion and fetal death	17 (2.7)
Short stature	17 (2.7)
Seizures	15 (2.4)
Skin manifestations	10 (1.6)
Others	267 (43.8)
Total	609 (100)

* Includes genetic retrospective and prospective counseling: consanguinity, fetal loss, repeat abortions, family history of birth defects, prenatal diagnosis;

† sensory-motor neuropathy, ataxia, and suspected Huntington’s disease.

NPDD: neuropsychomotor development delay.

Neurogenetic diseases, such as Huntington, Machado-Joseph, and Charcot-Marie-Tooth were among the diagnoses in adulthood. Approximately 40.0% of patients were under investigation at the time of the analysis.

Table 3 shows Mendelian inheritance was most often identified in the sample (17.8%; n=109), predominantly autosomal dominant, followed by autosomal recessive and X-linked. Chromosomal disorders and multifactorial inheritance totaled up 13.6% and 4.7% of cases, respectively. Inconclusive diagnosis was observed in 6.5% of patients, including individuals who did not return to the clinic for follow-up visits.

**Table 3:** Mode of inheritance of clinically diagnosed diseases of patients seen at the Medical Genetics Outpatient Clinic

Inheritance		n (%)
Mendelian		109 (17.8)
	Autosomal dominant	73 (11.9)
	Autosomal recessive	22 (3.6)
	X-linked	14 (2.2)
Under investigation		245 (40.2)
Chromosomal		83 (13.6)
Inconclusive*		40 (6.5)
Multifactorial		29 (4.7)
Others		9 (1.4)
Non-genetic		94 (15.4)
Total		609 (100)

* Including those who did not return to the clinic for follow-up visits.

**Figure 1:**
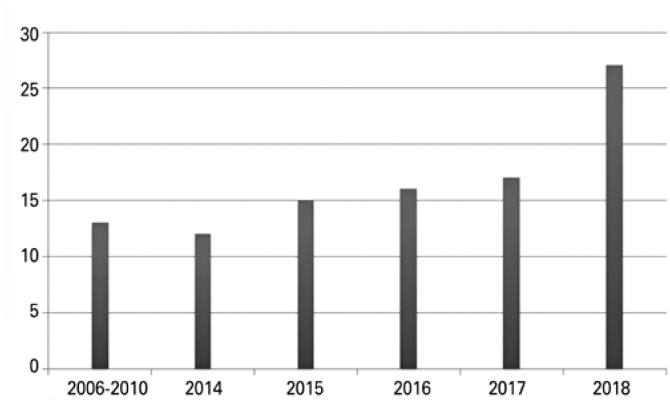
Genetic consultations requested

**Figure 2:**
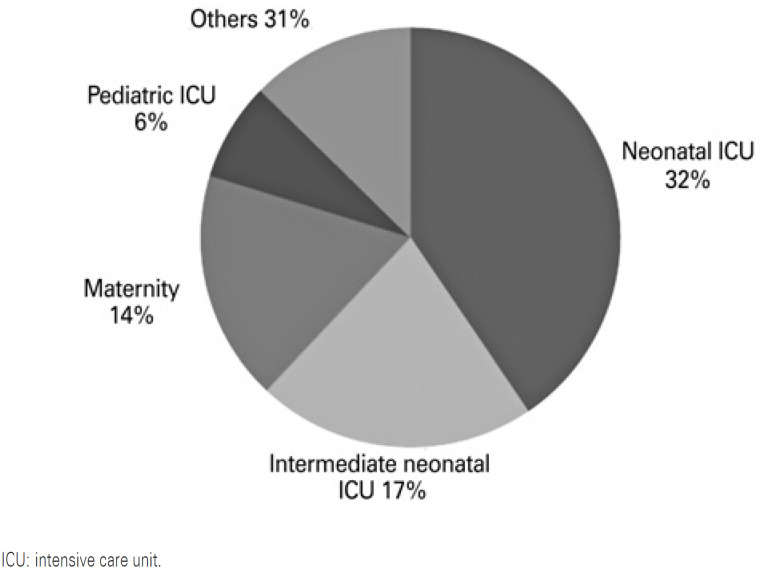
Units that requested genetic consulting

Genetic consultations, defined as genetic evaluations of patients admitted to the hospital at the request of other specialists, totaled up 100, in 12 years of study. Regarding distribution, 67.3% were male, 58.9% were aged zero to 7 days, 32.3% aged 8 days to 12 months, and 8.8% aged 1 to 5 years. Figure 1 shows this type of evaluation has been in growing demand, doubling the annual number of genetic consultations requested in the last 5 years. Figure 2 shows that despite the increased number of requests, they are concentrated in pediatric units. The main reason for consulting requests was “suspected genetic syndrome” (44.6%), which, in turn, was characterized by atypical facies, accompanied by dysmorphisms and/or malformations, appearing to be a classic syndrome or not. For 77.9% of evaluated children, some complementary tests were ordered, such as karyotype with G bands (44.0%), imaging exams, such as CT scan, ultrasound, MRI, or radiographs (22%), and analyses for inborn errors of metabolism (11.9%). No tests were ordered for 33.6% of children assessed. In 42.2% of children evaluated, a genetic diagnosis was made as follows: chromosomal (19.4%), monogenic (13.9%), multifactorial (8.9%), or environmental, such as that related to teratogenic mechanism (3.8%).

Among patients with chromosomal abnormalities, autosomal and sex chromosomal trisomy, deletions and derivative chromosomes were the most common findings. For about 31% of individuals evaluated, the causes of genetic etiology were excluded, ruling out a classic syndrome by the absence of clinical criteria after evaluation, or confirming other hypotheses, such as congenital infections, neonatal anoxia, and sequelae of prematurity or even family characteristics. The sum of these two groups, genetic and non-genetic, resulted in 73.4% of consultations with resolution during hospitalization, or by the first follow-up visit at the outpatient level. Among those evaluated, 26% were classified as inconclusive, including 4.7% of deaths, 14.3% who abandoned follow-up after hospital discharge, and 82.9% who continued in outpatient genetic care.

## DISCUSSION

The total number of outpatient visits in the period seems insignificant if we consider that, according to the prevalence of genetic diseases, roughly 14 thousand individuals in the municipality could be affected by such type of disease.^(^^9^^)^

The first consideration to be made is that this outpatient clinic is essentially an academic initiative, linked to a medical school. There is no contract with the Municipal Health Secretariat or the HU-FURG - the hospital only provides room for care, and it has not been accredited yet as a reference or specialized care service for rare diseases.

Another factor may be the long follow-up period of patients, given their needs and the lack of knowledge of other specialists about their diseases, making it impossible to have a greater number of discharges and, consequently, new consultations. Horovitz et al. described the “psychological bond” that occurs among parents and other professionals after delivery of genetic care.^(^^7^^)^

On the other hand, almost 80% of patients were from the city where the study was conducted, suggesting that there is a demand for genetic care. Approximately 9% of patients were referred from the private sector, and roughly 5% sought care spontaneously, again pointing to the need for this type of care. The low percentage of patients from rural areas suggests that, perhaps due to the inherent deficiencies and difficulties, access to the service is even more difficult for this population. Having a local service for the municipality can mean lower costs, because each patient to be taken to the capital city will usually need a companion, and several consultations and exams will be performed over the years. Additionally, since there is no commuting and waiting for evaluation in other centers, which are usually overloaded, it offers more agility to scheduling and performance of consultation, making diagnosis, treatment, and multidisciplinary follow-up faster, as already reported in other states.^(^^10^^−^^12^^)^

It is also important to remember that genetic care affects not only the individual, but also the family. In the case of hereditary diseases, the diagnosis may affect multiple individual and family decisions, including reproductive choices.^(^^5^^)^ There is also the psychological impact of confirming a disease, which may affect several people and generations.

Since most patients were children referred by their pediatricians or neurologists, the reasons for referral involved developmental delay, school difficulty, and behavioral changes. Such situations may be related to intellectual disability, which affects 1% to 3% of world population,^(^^13^^)^ and, in many cases, is genetic,^(^^14^^)^ as reported in this population. Similar data was also found at the Medical Genetics Outpatient Clinic of the *Universidade Federal de São Carlos* (UFSCar), which has also been active since 2006 and is another example of care provided outside large centers.^(^^10^^)^ The suspicion of autism spectrum disorder, also related to the same above-mentioned reasons for referral and present in 3.2% of cases, is expected, since the condition has had an increasing prevalence in recent years.^(^^11^^)^

Mendelian inheritance was the most frequent etiological diagnosis. According to the WHO,^(^^12^^)^ the global prevalence of all monogenic diseases is 10 per one thousand births. Such diseases may account for more than two-thirds of pediatric admissions and 40% of hospital admissions in some countries,^(^^15^^)^ which refers to the number of consultations requested by the pediatric units of HU-FURG, and the number of consultations referred by pediatricians. Furthermore, for these patients, considering the above, the availability of more sophisticated laboratory tools is important.

GTG karyotype is a useful test, but it is no longer the first choice in cases of intellectual disability. Some tests, such as array-CGH, recommended in cases of developmental delay, intellectual disability, and also in autism spectrum disorder,^(^^15^^)^ may elucidate many cases of the present study, providing appropriate genetic counseling and better management of the individuals.^(^^13^^,^^14^^)^ However, it should be noted that karyotyping was extremely useful in our outpatient clinic, because by diagnosing numerical and structural chromosomal abnormalities, it provided appropriate genetic counseling for the families.

Another aspect to consider is that care takes place in an academic environment, linked to the department of medical genetics and to residency in pediatrics, which allows proximity to such problems for future professional life, and may reflect in a greater number of referrals over time.

This study has several limitations, starting with its descriptive design. It also does not include socioeconomic variables, or provide the profile of patients, except for health issues. Another limitation is the large number of patients still under investigation, suggesting the need for greater resoluteness. The delay in performing tests, the availability of some only through research projects, and the difficult access to complementary evaluations and imaging exams may be contributing factors to these regarding outpatient consultations. On the other hand, the hospital consultations seem to be a useful tool during hospitalization, for they can increase diagnostic accuracy, and also reduce the time to initiate therapy, whether curative or palliative. In addition, they guide the family and professionals towards prognosis, and affect costs by avoiding unnecessary tests. Greater agility for collection of tests during hospitalization, as well as the availability of highly complex exams, are probably related to the shorter resolution time. Furthermore, the three-year interruption period may have caused losses, since it was necessary to restart the registration and tabulation of patient data at the medical genetics outpatient clinic.

## CONCLUSION

Within the scope of the Unified Health System, despite the need for expensive tests that are performed out of town, there is a positive impact because the outpatient clinic has gradually consolidated. With the centralization of the services, it receives patients from cities for which it is a reference, from Primary Healthcare Units, Family Health Units, and organizations that see people with disabilities. Thus, the order for unnecessary tests is avoided, health supervision for those affected is promoted, and complications are prevented. Genetic counseling and its aspects reach family members who would not have such guidance.

This type of service, provided at a medical school, is a teaching scenario and therefore, a training scenario, and may contribute in the long-term, to a greater professional interpellation and even to a greater number of medical geneticists who are physicians, decentralizing even more the service in genetics.
