# Supplementary material for: Medical care in clinical genetics: an experience of decentralization in southern Brazil
Source: Einstein (Sao Paulo). 2021 Jun 2;19:eAO5708. doi: 10.31744/einstein_journal/2021AO5708 (PMC8225263; doi:10.31744/einstein_journal/2021AO5708)
Supplement: Translation

# Translation: Atendimento em genética clínica: uma experiência de descentralização na região Sul do Brasil

**Authors:** Kevin Francisco Durigon Meneghini, Simone de Menezes Karam, Victor Francis Pereira Madruga, Andrea Schulz Silva Ungaretti, Eduarda Cecilia Pinguello, Rafaely Severo

**Affiliations:** 1 Rio Grande do SulRS Brasil Hospital Universitário Dr. Miguel Riet Corrêa Jr., Faculdade de Medicina, Universidade Federal do Rio Grande, Rio Grande do Sul, RS, Brasil.

**Keywords:** Genética médica, Pesquisa sobre serviços de saúde, Epidemiologia, Saúde Pública, Hospitais universitários

## Abstract

**Objetivo:**

Descrever a população atendida em um ambulatório de genética, em uma cidade de médio porte, em relação a diagnóstico, tipo de herança e impacto local do atendimento.

**Métodos:**

Foram revisados os prontuários e formulários do serviço de genética entre 2006 e 2018. As variáveis analisadas foram idade, sexo, procedência e atual residência, motivo de referência/consultoria, profissional que encaminhou, diagnóstico final, exames complementares e seus resultados.

**Resultados:**

Foram atendidos 609 pacientes, 65,9% com idade de zero a 12 anos. Houve suspeita de síndromes genéticas em 15,1%, e 11% apresentaram atraso no desenvolvimento. Distúrbios neurogenéticos destacaram-se entre os adultos. A herança mendeliana teve maior frequência (17,8%). As solicitações de consultorias genéticas duplicaram nos últimos 5 anos, sendo 44,4% por “suspeita de síndrome genética”.

**Conclusão:**

As consultorias genéticas mostraram-se ferramenta importante em nível hospitalar, reduzindo o tempo de espera até a instituição terapêutica, minimizando custos potenciais associados e norteando os familiares. O atendimento ambulatorial ofereceu diagnóstico e aconselhamento genético para usuários da cidade e região, diminuiu custos, além de propiciar um ambiente de ensino em genética médica.

## INTRODUÇÃO

A portaria 199/2014 do Ministério da Saúde instituiu, no âmbito do Sistema Único de Saúde (SUS), a política de atenção às doenças raras (DR), prevendo a estruturação do atendimento desde a prevenção até a reabilitação. São consideradas DR aquelas que afetam 65/100 mil indivíduos, de acordo com a Organização Mundial da Saúde (OMS).^(^^1^^)^ As doenças genéticas podem acometer até 73 a cada mil indivíduos.^(^^2^^)^ As anomalias congênitas, determinadas majoritariamente por causas genéticas, afetam 5% dos recém-nascidos.^(^^3^^)^ Tais condições causam grande impacto aos indivíduos afetados e a suas famílias, tanto por levarem a condições físicas debilitantes, quanto pelo fato de acometerem vários membros de uma mesma família, resultando em incapacidade, desemprego e discriminação.^(^^4^^)^

A busca por um diagnóstico definitivo é, com frequência, muito longa, acarretando em ainda mais sofrimento.^(^^5^^)^ Em contraste, o Brasil conta com 305 médicos especialistas em genética médica, o equivalente a uma razão de 0,15 especialista por 100 mil habitantes, sendo 60% deles atuantes na Região Sudeste.^(^^6^^)^ Na maioria dos Estados, os geneticistas ainda atuam nas capitais.^(^^7^^)^

## OBJETIVO

Descrever a população atendida em um ambulatório de genética, em uma cidade de médio porte, em relação a diagnóstico, tipo de herança e impacto local do atendimento.

## MÉTODOS

Foi realizado um estudo transversal, retrospectivo, baseado na revisão de prontuários do Ambulatório de Genética Médica, localizado em Rio Grande, e nos formulários de consultoria em genética, entre 2006 e 2018, excluindo-se um período de 3 anos (2011, 2012 e 2013), totalizando 10 anos de atendimento. No período de afastamento, alguns pacientes receberam alta ou foram encaminhados para outro serviço.

Rio Grande, local do presente estudo, é um município com 2.709km^2^ localizado na metade sul do Estado do Rio Grande do Sul, e distante 350km da capital Porto Alegre, com cerca de 208 mil habitantes, sendo 96% deles residentes na área urbana.^(^^8^^)^ A economia tem como principal atividade a portuária, além da pesca e do refino de petróleo.^(^^8^^)^ Na cidade, situa-se a Universidade Federal do Rio Grande (FURG), cujo Hospital Universitário Dr. Miguel Riet Corrêa Júnior (HU-FURG), com atendimento exclusivamente pelo SUS, é referência para 22 cidades de sua região. O HU-FURG disponibiliza atendimento em genética desde 2006.

A população-alvo foi composta por crianças e adultos que consultaram no ambulatório durante o referido período. Para a revisão, foi utilizada uma ficha padronizada contendo as seguintes variáveis: idade, sexo, procedência do paciente, local atual de residência, motivo de referência ou de consultoria hospitalar, profissional responsável pela referência, diagnóstico final, exames necessários para a investigação e seus resultados. Todos os dados foram tabulados no programa Excel e classificados quanto ao tipo de herança e diagnóstico conclusivo ou não. Foram considerados em investigação pacientes que fizeram pelo menos uma consulta e, até o momento de finalizar a coleta de dados, ainda aguardavam exames complementares e/ou outras avaliações especializadas para conclusão e indivíduos não afetados e que buscaram aconselhamento genético pré-concepcional por idade parental, consanguinidade ou outros motivos. A classificação “não genética” foi dada aos casos em que havia suspeita de doença genética, mas nos quais ela não se confirmou após investigação clínica e laboratorial. Os dados foram coletados por alunos de graduação e revisados e analisados em conjunto com a investigadora principal. O Ambulatório de Genética Médica possui um protocolo específico de primeira consulta, onde foram registradas a anamnese e a história familiar com heredograma, contemplando pelo menos três gerações, história pré-natal, do parto e do período neonatal, além de registro de vacinação, dos marcos do desenvolvimento, do uso de medicações e dos exames laboratoriais e/ou de imagem já realizados. Foram realizados exame físico geral, antropometria e exame dismorfológico. Se houvesse indicação, procedeu-se a exame neurológico.

De acordo com o que foi confirmado ao final da consulta ou no decorrer da investigação, as consultas subsequentes foram registradas em protocolos de seguimento, por exemplo, para síndrome de Turner, síndrome de Down, ataxia cerebelar dominante, facomatoses, deficiência intelectual, dentre outras. Tais protocolos contemplam a evolução do indivíduo e a orientação antecipatória. No período do estudo, o ambulatório dispunha dos seguintes exames para investigação: radiografias, ultrassonografia, ecocardiograma, eletrocardiograma, eletroencefalografia e tomografia no próprio HU-FURG, além do laboratório de análises clínicas. Cariótipo dos cromossomos por bandamento G (GTG) e reação em cadeia de polimerase (PCR) para algumas mutações, eram realizadas em laboratório conveniado com o hospital. Erros inatos do metabolismo (triagem metabólica e dosagem de atividade enzimática) foram verificados por meio da Rede EIM Brasil), no Hospital de Clínicas de Porto Alegre (HCPA) e na Universidade Federal do Rio Grande do Sul (UFRGS). Exames de citogenética mo­lecular (hibridização *in situ* por fluorescência – FISH – e hibridização genômica comparativa baseada em microarranjos (*array*-CGH) foram feitos por meio de colaboração temporária em projeto de pesquisa com a Rede Brasileira de Referência e Informação em Síndromes de Microdeleção (REDEBRIM/HCPA/UFRGS). Não foi realizado sequenciamento de nova geração, pois não estava disponível no sistema público.

A conduta inicial baseou-se nas informações clínicas, e as hipóteses foram investigadas até que os meios disponíveis, quer em nível local, quer por meio de colaborações de outros centros, fossem esgotados. Quando a suspeita de um quadro genético persistiu, o paciente continuou em avaliação clínica semestral ou anual.

Em dois diferentes períodos e novamente desde início de 2018, os exames de cariótipo e as técnicas moleculares de PCR não foram mais disponibilizados. A investigação de câncer familial nunca foi realizada. Casos que preenchiam critérios de uma síndrome de predisposição hereditária ao câncer foram encaminhados ao Ambulatório de Triagem do Hospital de Clínicas de Porto Alegre, por meio da Secretaria Municipal de Saúde.

A mesma ficha padronizada para avaliar as consultas ambulatoriais foi utilizada para avaliar as consultorias em genética clínica no mesmo período. O termo “consultoria” refere-se à avaliação genética realizada em pacientes internados no HU-FURG, solicitada por outro especialista. Para a condução, o raciocínio clínico e os exames disponíveis já descritos foram as ferramentas utilizadas.

O projeto foi aprovado pelo Comitê de Ética em Pesquisa na Área da Saúde (CEPAS-FURG), parecer 4212100, CAAE: 93280418.3.0000.5324.

## RESULTADOS

No período estudado, 609 pacientes consultaram o Ambulatório de Genética Médica. Três quartos deles (78,6%) eram provenientes da cidade onde se localizava o serviço, 98,4% eram moradores da zona urbana, e 65,9% estavam na faixa de zero a 12 anos de idade. Mais da metade era do sexo feminino (Tabela 1). Parte dos pacientes foi encaminhada de consultórios privados, sendo o neurologista (n=25) o especialista que mais encaminhou, seguido do pediatra (n=24) e do ginecologista-obstetra (n=7).

**Tabela 1:** Procedência, idade e sexo dos pacientes

Procedência		n (%)
Município de Rio Grande		479 (78,6)
	Outros	130 (21,4)
Origem do encaminhamento		
	Pediatra	111 (18,2)
	Neurologista	58 (9,5)
	Consultório privado	56 (9,1)
	APAE	34 (6,6)
	UBS e UBSF	31 (5,0)
	Espontâneo/Familiar	29 (4,7)
	Outro geneticista	6 (0,9)
	Outros	284 (46,6)
Idade, ano		
	<1	134 (22,0)
	1-4	107 (17,5)
	5-11	161 (26,4)
	12-19	86 (14,1)
	≥20	115 (18,8)
	Não informado	5 (0,8)
Sexo		
	Feminino	333 (54,6)
	Masculino	276 (45,4)
Total		609 (100)

APAE: Associação de Pais e Amigos dos Excepcionais; UBS: Unidade Básica de Saúde; UBSF: Unidade Básica de Saúde da Família.

Em relação às referências para consulta genética (Tabela 2), destacou-se a “suspeita de síndrome não especificada” (15,1%), que se referia a encaminhamentos sem descrição de detalhes relativos a desenvolvimento, comportamento ou alterações morfológicas, sem apontar a suspeita de uma síndrome clássica. Em segundo lugar, estava o atraso no desenvolvimento neuropsicomotor associado ou não a dismorfias e/ou alterações de comportamento (11%). Aproximadamente 6% das referências pertenciam ao grupo de aconselhamento genético, no qual foram incluídos aconselhamento retrospectivo e prospectivo.

**Tabela 2:** Motivos mais frequentes de referência para consulta genética

Motivos	n (%)
Suspeita de síndrome não especificada	92 (15,1)
ADNPM associado ou não a dismorfias e/ou a alterações de comportamento	67 (11,0)
Aconselhamento genético*	35 (5,7)
Dificuldade escolar isolada ou associada	31 (5,0)
Suspeita de transtorno do espectro autista	20 (3,2)
Suspeita de síndrome de Down	20 (3,2)
Suspeita de doença neurogenética†	18 (2,9)
Abortamento de repetição e óbito fetal	17 (2,7)
Baixa estatura	17 (2,7)
Crises convulsivas	15 (2,4)
Manifestações dermatológicas	10 (1,6)
Outros	267 (43,8)
Total	609 (100)

* Inclui aconselhamento genético retrospectivo e prospectivo: consanguinidade, perdas fetais, abortamentos de re­petição, história familiar de defeitos congênitos, diagnóstico pré-natal;

† neuropatia sensitivo-motora, ataxia e suspeita de doença de Huntington.

9 ADNPM: atraso no desenvolvimento neuropsicomotor.

Dentre os diagnósticos na idade adulta, estavam doenças neurogenéticas, como as de Huntington, Machado-Joseph e Charcot-Marie-Tooth. Cerca de 40,0% dos pacientes estavam sob investigação no momento da análise.

A tabela 3 mostra que a herança identificada com maior frequência na amostra foi a mendeliana, com 17,8% (n=109), predominando a autossômica dominante, se­guida por autossômica recessiva e ligada ao X. Cromossomopatias e herança multifatorial tota­lizaram 13,6% e 4,7%, respectivamente. Dentre os 6,5% de pacientes com diagnóstico inconclusivo, incluíram-se indivíduos que não retornaram ao ambulatório para as consultas de seguimento.

**Tabela 3:** Modo de herança das doenças diagnosticadas clinicamente dos pacientes do ambulatório de Genética Médica

Herança das doenças		n (%)
Mendeliana		109 (17,8)
	Autossômica dominante	73 (11,9)
	Autossômica recessiva	22 (3,6)
	Ligada ao X	14 (2,2)
Em investigação		245 (40,2)
Cromossômico		83 (13,6)
Inconclusivo*		40 (6,5)
Multifatorial		29 (4,7)
Outros		9 (1,4)
Não genético		94 (15,4)
Total		609 (100)

* Contêm os que não retornaram ao ambulatório para as consultas de seguimento.

Consultorias genéticas, definidas como avaliações genéticas de pacientes em internação hospitalar a pedido de outros especialistas, totalizaram cem nos 12 anos do estudo. Em relação à distribuição, 67,3% eram do sexo masculino, 58,9% tinham entre zero e 7 dias de vida, 32,3% entre 8 dias e 12 meses de idade e 8,8% entre 1 e 5 anos de idade. Na figura 1, observa-se que esse tipo de avaliação vinha sendo mais requisitado, duplicando o número anual de consultorias genéticas solicitadas nos últimos 5 anos. Na figura 2, nota-se que, apesar do aumento no número de solicitações, estas concentram-senas unidades pediátricas. O principal motivo para solicitação de consultoria foi a “suspeita de síndrome genética” (44,6%), a qual, por sua vez, foi caracterizada por face atípica, acompanhada de dismorfias e/ou malformações, aparentando síndrome clássica ou não. Para 77,9% dos avaliados, solicitou-se algum exame completar: cariótipo com bandas G (44,0%), exa­mes de imagem, como tomografia computadorizada, ultrassonografia, ressonância magnética ou radiografias (22%), e testes para análise de erros inatos do metabolismo (11,9%). Para 33,6% das crianças avaliadas, não foram solicitados exames. Em 42,2% dos avaliados, concluiu-se por um diagnóstico genético: cromossômico (19,4%), monogênico (13,9%), multifatorial (8,9%) ou ambiental, como o relacionado a teratógenos (3,8%). Dentre os pacientes com alterações cromossômicas, trissomias dos autossomos e dos cromossomos sexuais, deleções e cromossomos derivativos foram os resultados mais comuns. Para cerca de 31% dos indivíduos avaliados, foram excluídas causas de etiologia genética, descartando-se uma síndrome clássica, pela ausência de critérios clínicos, após avaliação, ou confirmando-se outras hipóteses, como infecções congênitas, anóxia neo­natal e sequelas da prematuridade, ou mesmo características familiares. A soma desses dois grupos, genético e não genético, resultou em 73,4% das consultorias com resolução durante a internação ou até a primeira consulta de revisão em nível ambulatorial. Dentre os avaliados, 26% foram classificados como inconclusivos, incluindo 4,7% de óbitos, 14,3% que abandonaram o acompanhamento após a alta hospitalar e 82,9% que continuaram em atendimento genético ambulatorial.

**Figura 1:** Consultorias genéticas solicitadas

**Figura 2:** Unidades que solicitaram consultorias genéticas

## DISCUSSÃO

O total de atendimentos ambulatoriais no período parece pouco expressivo se for considerado que, de acordo com a prevalência de doenças genéticas, cerca de 14 mil indivíduos no município poderiam ser afetados por alguma doença desse tipo.^(^^9^^)^

A primeira consideração a ser feita é que esse ambulatório é uma iniciativa essencialmente acadêmica, vinculada a uma faculdade de medicina. Não há contratualização com a Secretaria Municipal de Saúde e nem com o HU-FURG – o hospital apenas cede o local para o atendimento, e ele próprio ainda não é credenciado como um serviço de referência ou de atenção especializada para DR.

Outro fator pode ser o longo período de acompanhamento dos pacientes, dadas as necessidades que apresentam e o desconhecimento de outros especialistas sobre suas moléstias, impossibilitando um número maior de altas e, consequentemente, de consultas novas. Horovitz et al., descrevem o “vínculo psicológico” que ocorre após a obtenção do atendimento em genética, tanto por parte dos pais como de outros profissionais.^(^^7^^)^

Por outro lado, quase 80% dos atendidos eram da própria cidade, sugerindo que existe demanda por atendimento genético. Cerca de 9% dos pacientes foram encaminhados do nível privado e em torno de 5% procuraram o atendimento espontaneamente, acenando novamente para a necessidade desse tipo de atendimento. O baixo percentual de pacientes oriundos da zona rural sugere que, talvez pelas deficiências e dificuldades inerentes, o acesso ao serviço seja ainda mais difícil para essa população. Ter um atendimento local, para o município, pode significar diminuir custos, pois cada paciente a ser levado para consulta na capital necessitará quase sempre de acompanhante, e serão realizadas várias consultas e coletas de exames ao longo de anos. Além disso, por não haver deslocamento e espera por avaliação em outros centros, usualmente já sobrecarregados, oferece mais agilidade ao agendamento e a realização da consulta, tornando o diagnóstico, o tratamento e o acompanhamento multidisciplinar mais rápidos, como já relatado em outros estados.^(^^10^^−^^12^^)^

É preciso lembrar, ainda, que o atendimento em genética impacta não somente no indivíduo, mas também em sua família. Em se tratando de doenças hereditárias, o diagnóstico pode repercutir em múltiplas decisões individuais e familiares, incluindo escolhas reprodutivas.^(^^5^^)^Há ainda o impacto psicológico da confirmação de uma doença, que pode acometer várias pessoas e diversas gerações.

Dado que a maior parte dos pacientes era de criança encaminhada por seus pediatras ou neurologistas, os motivos de referência incluíram atraso no desenvol­vimento, dificuldade escolar e alterações de comporta­mento. Tais situações podem estar relacionadas à deficiência intelectual, que acomete 1% a 3% da população mundial^(^^13^^)^ e que, em muitos casos, é genética,^(^^14^^)^ como visto nesta população. Dado semelhante também foi encontrado no Ambulatório de Genética Médica da Universidade Federal de São Carlos (UFSCar), em atividade também desde 2006 e que consiste em outro exemplo de atendimento fora dos grandes centros.^(^^10^^)^ A suspeita de transtorno do espectro autista, também relacionada aos mesmos motivos de referência mencionados e presente em 3,2% dos encaminhamentos é esperada, já que a condição tem tido prevalência crescente nos últimos anos.^(^^11^^)^

Herança mendeliana foi diagnóstico etiológico mais frequente. De acordo com a OMS,^(^^12^^)^ a prevalência global de todas as doenças monogênicas é de dez a cada mil nascimentos. Tais doenças podem somar mais de dois terços das admissões pediátricas e 40% das admissões hospitalares em alguns países,^(^^15^^)^ o que remete ao número de consultorias solicitadas pelas unidades pediátricas do HU-FURG e ao número de consultas referenciadas por pediatras. Ainda, para esses pacientes, considerando o que já foi exposto, é importante a disponibilidade de ferramentas laboratoriais mais sofisticadas.

Ainda que o cariótipo GTG seja um exame útil, nos casos de deficiência intelectual, ele já não é mais a primeira escolha. Exames como o *array*-CGH, recomendado em casos de atraso no desenvolvimento, deficiência intelectual e também no transtorno do espectro autista,^(^^15^^)^ talvez possam elucidar muitos casos do presente estudo, propiciando adequado aconselhamento genético e melhor manejo dos indivíduos.^(^^13^^,^^14^^)^ Contudo, é preciso ressaltar que o cariótipo foi extremamente útil em nosso ambulatório, pois, ao diagnosticar alterações cromossômicas numéricas e estruturais, propiciou aconselhamento genético adequado para as famílias.

Outro aspecto a considerar é que os fatos de o atendimento acontecer em ambiente acadêmico e estar vinculado à disciplina de genética médica e à residência em pediatria permitem proximidade com tais problemas para a vida profissional futura, podendo refletir em maior número de referências ao longo do tempo.

Este estudo apresenta várias limitações, a começar pelo delineamento descritivo e por não incluir variáveis socioeconômicas, não permitindo conhecer o perfil dos atendidos, exceto no que diz respeito à sua saúde. Outra limitação é o grande número de pacientes ainda em investigação, sugerindo a necessidade de maior resolutividade. A demora na realização de exames, a disponibilidade de alguns apenas por meio de projetos de pesquisa e o difícil acesso a avaliações complementares e a exames de imagem podem ser fatores contribuintes para isso no que tange às consultas ambulatoriais. As consultorias hospitalares, por sua vez, parecem ser uma ferramenta útil na internação, pois, na medida em que podem aumentar a precisão diagnóstica, também podem diminuir o tempo até a instituição da terapêutica,seja ela curativa ou paliativa, assim como nortear a família e os profissionais em termos de prognóstico e, ainda, impactar nos custos evitando exames desnecessários. A maior agilidade para a coleta de exames durante a internação, bem como a disponibilidade de exames de alta complexidade, provavelmente está relacionada ao tempo mais curto de resolução. Ademais, o período de interrupção de 3 anos pode ter acarretado prejuízo, uma vez que houve a necessidade de recomeçar o registro e tabelamento dos dados dos pacientes do ambulatório de genética médica.

## CONCLUSÃO

No âmbito do Sistema Único de Saúde, apesar da necessidade de exames caros e realizados fora da cidade, acredita-se haver um impacto positivo, pois, aos poucos, o ambulatório consolida-se. Com a centralização do atendimento, ele recebe pacientes de cidades para as quais é referência, de Unidades Básicas de Saúde, Unidades Básicas de Saúde da Família e instituições de atendimento a pessoas com deficiências. Assim, evita-se a solicitação de exames desnecessários, promove-se a supervisão de saúde para os afetados e previnem-se complicações. O aconselhamento genético e seus aspectos, desse modo, chega a familiares que antes não teriam tal orientação.

Esse tipo de atendimento, a partir de uma faculdade de medicina, é cenário de ensino e, portanto, de formação, podendo, em longo prazo, contribuir para maior interpelação profissional e até para um maior número de médicos geneticistas, descentralizando ainda mais o atendimento em genética.
